# Supplementary material for: Deposit-feeding worms control subsurface ecosystem functioning in intertidal sediment with strong physical forcing
Source: PNAS Nexus. 2022 Aug 18;1(4):pgac146. doi: 10.1093/pnasnexus/pgac146 (PMC9802194; doi:10.1093/pnasnexus/pgac146)
Supplement: pgac146_Supplemental_Files [file pgac146_supplemental_files.zip › PNASNEXUS-PNASNEXUS-2022-00214-T-s02.docx]

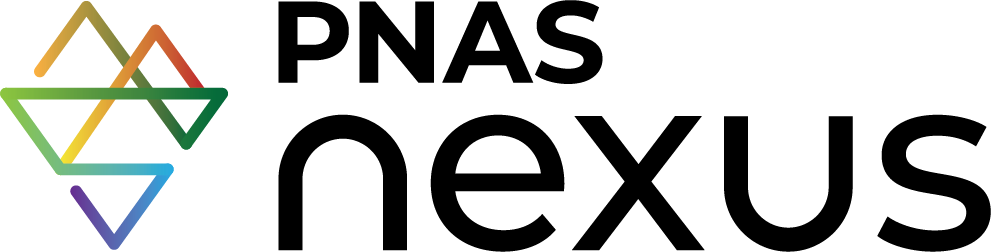


**Supplementary Materials for**

Deposit-feeding worms control subsurface ecosystem functioning in intertidal sediment with strong physical forcing

Longhui Deng,^1,^ ^†*^ Christof Meile,^2^ Annika Fiskal,^1, ‡^ Damian Bölsterli,^1^ Xingguo Han,^1,^ ^§^ Niroshan Gajendra,^1^ Nathalie Dubois,^3,4^ Stefano M. Bernasconi,^4^ Mark A. Lever^1*^

^1^ Institute of Biogeochemistry and Pollutant Dynamics, ETH Zürich, 8092 Zürich, Switzerland.

^2^ Department of Marine Sciences, University of Georgia, Athens, GA 30602, USA.

^3^ Surface Waters Research-Management, Eawag, Swiss Federal Institute of Aquatic Science and Technology, 8600 Dübendorf, Switzerland.

^4^ Department of Earth Sciences, ETH Zürich, 8092 Zürich, Switzerland.

Present addresses:

^†^ School of Oceanography, Shanghai Jiao Tong University, Shanghai 200030, China.

^‡^ Department of Microbial Ecology, German Federal Institute of Hydrology (BfG), Am Mainzer Tor 1, 56068, Koblenz, Germany.

^§^ Forest Soils and Biogeochemistry, Swiss Federal Institute for Forest, Snow and Landscape Research WSL, 8903 Birmensdorf, Switzerland.

^*^**Correspondence:** Longhui Deng (longhui.deng@sjtu.edu.cn), Mark Alexander Lever ([mark.lever@usys.ethz.ch](mailto:mark.lever@usys.ethz.ch))

**Keywords:** Bioturbation, hydrodynamics, carbon cycling, community assembly, organismal networks

**Supplementary Text**

**Modeled rates of porewater exchange by physical forcing and bioirrigation**

Porewater exchanges due to physical mixing ($\alpha_{P}$) and bioirrigation ($\alpha_{B})$were defined as the non-local transport of fluids between sediments and overlying water as a function of sediment depth ($\alpha_{P}$*(x)*, $\alpha_{B}$*(x))*. To implement the simulations, several assumptions that are built on our experimental design and observations in the field were made: 1) physical mixing is intrinsically tied to the sediment surface; 2) the defaunated and refaunated plots are subject to identical impact of physical mixing due to the close vicinity of both plots; 3) rate profiles are similar in defaunated and refaunated plots due to the same initial homogenization (1). The general equation for the modeling is:

$\phi\frac{\partial C}{\partial t}=\frac{\partial}{\partial x}\left( \phi D\frac{\partial C}{\partial x} \right)+R+\alpha_{P}\phi\left( C_{0}-C \right)+\alpha_{B}\phi\left( C_{0}-C \right)$ (S1)

where $\phi$ is porosity (set to 0.65), *D* is the *in situ* diffusion coefficient corrected for tortuosity, $D={D_{mol}}/\left( 1-2ln\left( \phi\right) \right)$ (2), *R* is the species-specific net reaction rate, and *C_0_* is the concentration in overlying water.

Decreases in physical porewater mixing intensity with depth were represented by the function $\alpha_{P}(x)={\alpha_{P,0}}/\left( 1+e^{\alpha_{P,1}\left( x-\alpha_{P,2} \right)} \right)$, which reflects the fact that physical mixing is the result of external forcing (e.g. waves) from overlying water (e.g. 3). We chose a function in which the three parameters (a_P,0-2_) adjust the magnitude, shape and depth of physically induced mixing to match the measured porewater profiles.

The reaction rate profile was assumed to vary monotonously with depth, $R(x)={r_{0}}/\left( 1+e^{r_{1}\left( x-r_{2} \right)} \right)$. Such a profile is able to reflect either a near constant rate of mineralization with depth in mixed surface sediments, or a decrease in organic matter mineralization with depth as would be expected if reactive organic matter is newly added to the sediment surface (e.g. 4). The three parameters r_0-2_ together determine the magnitude, shape, and decrease of the rate profile with depth.

The bioirrigation profile shown in Figure 1C was set to mimic the behavior of lugworms that inject water into their J-shaped, dead-end burrows (e.g. 5). The injection depth was set to the average living depth of *A. pacifica* in the study area (~16 cm). Bioirrigation was assumed to decrease toward the sediment surface and be minimal below the living depth (5, 6).

Depth profiles of DIC production rate R(x), physical mixing coefficients $\alpha_{P}$(x), and bioirrigation coefficients $\alpha_{B}$(x) were fitted to measured DIC concentration profiles. DIC was used as a parameter as its concentrations are mainly controlled by organic matter degradation processes and physical or biological transport processes in temperate intertidal sediments (e.g. 7, 8). First, physical mixing and net DIC production profiles were estimated in the defaunated treatment (i.e. $\alpha_{B}$ = 0; R(x) and $\alpha_{P}$(x)). Using the initial DIC profile (T1) as starting conditions, the parameters (r_0-2_; $\alpha_{P}$_,0-2_) were adjusted to fit the DIC concentration profile measured 2 weeks later (T2). Next, based on the physical mixing and rate profiles determined in the defaunated plot, we estimated the bioirrigation coefficient profile to approximate the measured concentration profiles in the refaunated plot.

The quality of the fit was assessed visually, and mixing intensities at the low end of the range providing a good fit were chosen (Fig. S13). This conservative estimate was used because the parameterization is not unique, i.e., any sufficiently intense mixing can reproduce the observed constant DIC concentration in surficial sediments (0-5 cm) which match those of overlying water. Therefore, the derived mixing rates in surficial sediments might be underestimates. Notably, however, this does not affect our main finding that significant porewater mixing by the lugworms in subsurface sediments below the zone of physical mixing is required to explain the observations.

Finally, the rate and mixing profiles determined from matching the DIC profiles were validated by simulating the sulfate concentration profiles using the same physical and biological mixing profiles, and using a DIC production to sulfate consumption stoichiometry of 2:1 (i.e. assuming that dissimilatory sulfate reduction is the dominant mineralization process below the depth of measured O_2_ penetration). We observe a good match with measured sulfate concentrations (Fig. S13), which indicates that the derived mixing and rate profiles are reasonable estimates.

**Modeled rates of sediment mixing by physical and biological forcing**

The timescale (2 weeks) of the manipulation experiments turned out to be too short to observe significant changes in distributions of sediment particles. Solid-phase mixing was thus estimated based on vertical profiles of chlorophyll *a* and pheopigment contents in natural sediments of bioturbated and non-bioturbated sediments (as shown in Fig. 2). In the bioturbated sediments, rates of total sediment mixing (D_T_) include mixings from both physical (D_P_) and biological mixings (D_B_), while in the non-bioturbated sediments, D_T_ includes mainly physical mixing (D_P_). Sediment mixing was thus modeled as:

$\frac{\partial C}{\partial t}=\frac{\partial}{\partial x}\left( D_{T}\frac{\partial C}{\partial x} \right)-k_{C}C$ (S2)

$\frac{\partial P}{\partial t}=\frac{\partial}{\partial x}\left( D_{T}\frac{\partial P}{\partial x} \right)+k_{C}C-k_{P}P$ (S3)

$D_{T}=D_{B}+D_{P}$ (S4)

where C is the content of chl *a*, P is the content of pheopigments, k_C_ and k_P_ are the rate constants for the breakdown of chl *a* and pheopiments, respectively. k_C_ is set to 0.04 d^-1^ (average value from different literature sources 9-11). D_T_ is a mixing coefficient for total mixing, D_P_ and D_B_ are the mixing coefficients for physical and biological mixings, respectively. Given the negligible impact of macrofaunal bioturbation in non-bioturbated sediments, D_B_ was set to zero to solve the term for D_P_ in these (equation S4). The same D_P_ was then applied to bioturbated sediments to solve for D_B_.

We divide the sediment column into three layers (0-12.5, 12.5-25 and >25 cm; see Results of main text for details), each with a constant value of D_T_. The resulting analytical solutions for the concentration profiles in layer *i* are:

$C\left( x \right)=M_{1,i}exp\left( \sqrt{{k_{C}}/{D_{T,i}}}x \right)-M_{2,i}exp\left( -\sqrt{{k_{C}}/{D_{T,i}}}x \right)$ (S5)

and

$P\left( x \right)=N_{1,i}exp\left( \sqrt{{k_{P}}/{D_{T,i}}}x \right)-N_{2,i}exp\left( -\sqrt{{k_{P}}/{D_{T,i}}}x \right)+\frac{k_{C}}{k_{P}}C$ (S6)

The constants M_j,j_ and N_j,i_ were determined by the boundary conditions including the measured concentrations at the top (1 cm), depths between layers (i.e. 12.5 and 25 cm), flux continuation, and no-gradient conditions at infinite depth. We applied a k_P_ value of 0.015 d^-1^ that was measured in sandy sediments of an adjacent coastal location (Dabob Bay, Washington, USA; 12) for the non-bioturbated sediments. For the bioturbated sediments, however, this relatively low k_p_ did not reflect the fast removal and consequently low concentrations of pheopigments. A higher k_p_ value of 0.03 d^-1^ that approximates the rate constant of chl *a* was therefore applied for bioturbated sediments (value measured in coastal sandy/silty sediments that were subject to hydrodynamic forcing and bioturbation, Long Island Sound; 9). We then optimized the D_T_ value in each layer *i*. The parameterization we applied generally resulted in good fits for all data of chl *a*, pheopigments, as well as freshness index across the three different time points (Fig. S14).

**Supplementary Figures and Tables**


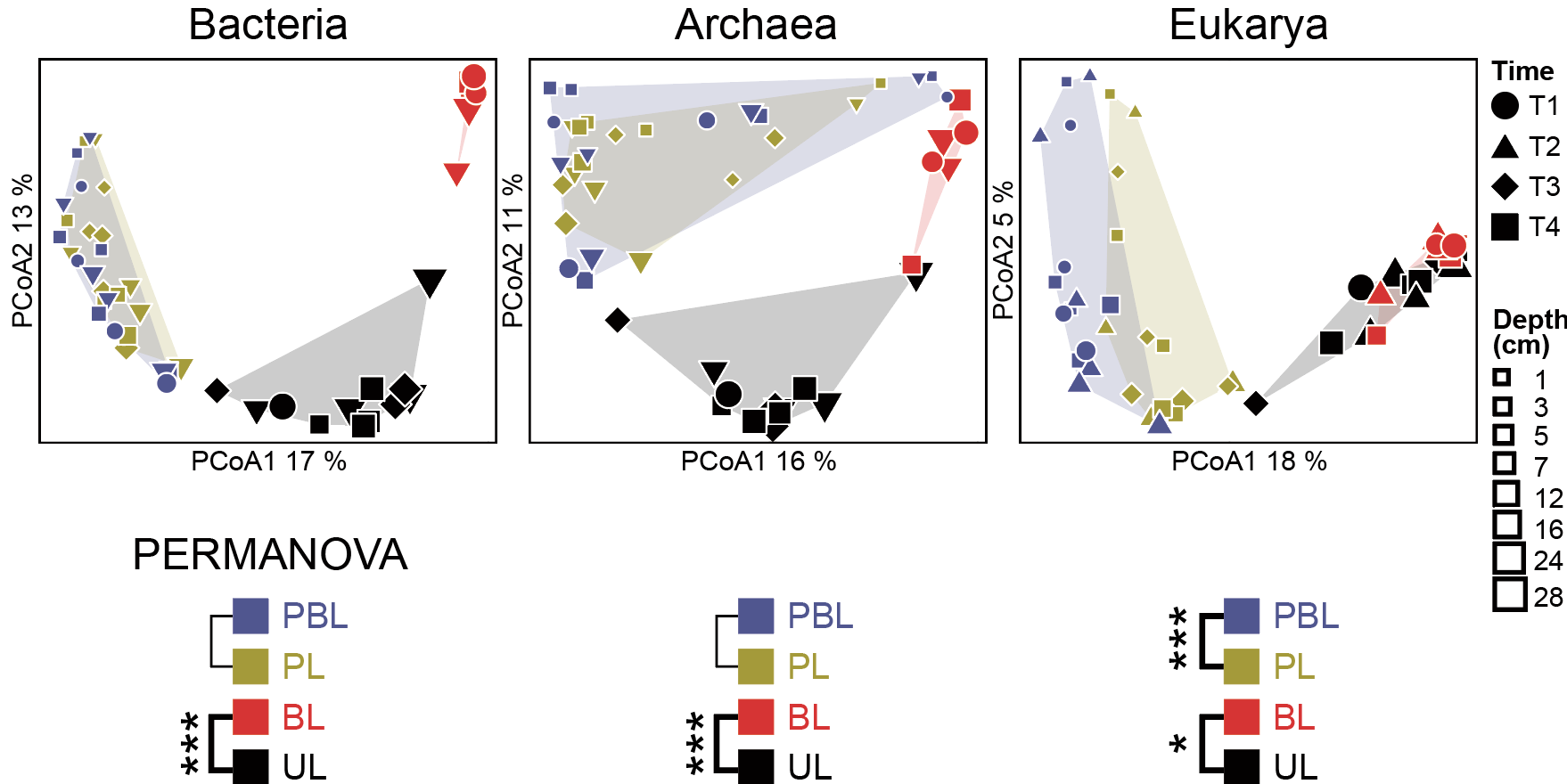


**Fig. S1.** Principal Coordinates Analyses (PCoA) based on unweighted Unifrac distance and PERMANOVA tests on bacterial, archaeal, and eukaryotic communities. In the calculation of community dissimilarity, unweighted Unifrac algorithm treats each taxon equally and is thus more sensitive at detecting differences in communities among low-abundance taxa.


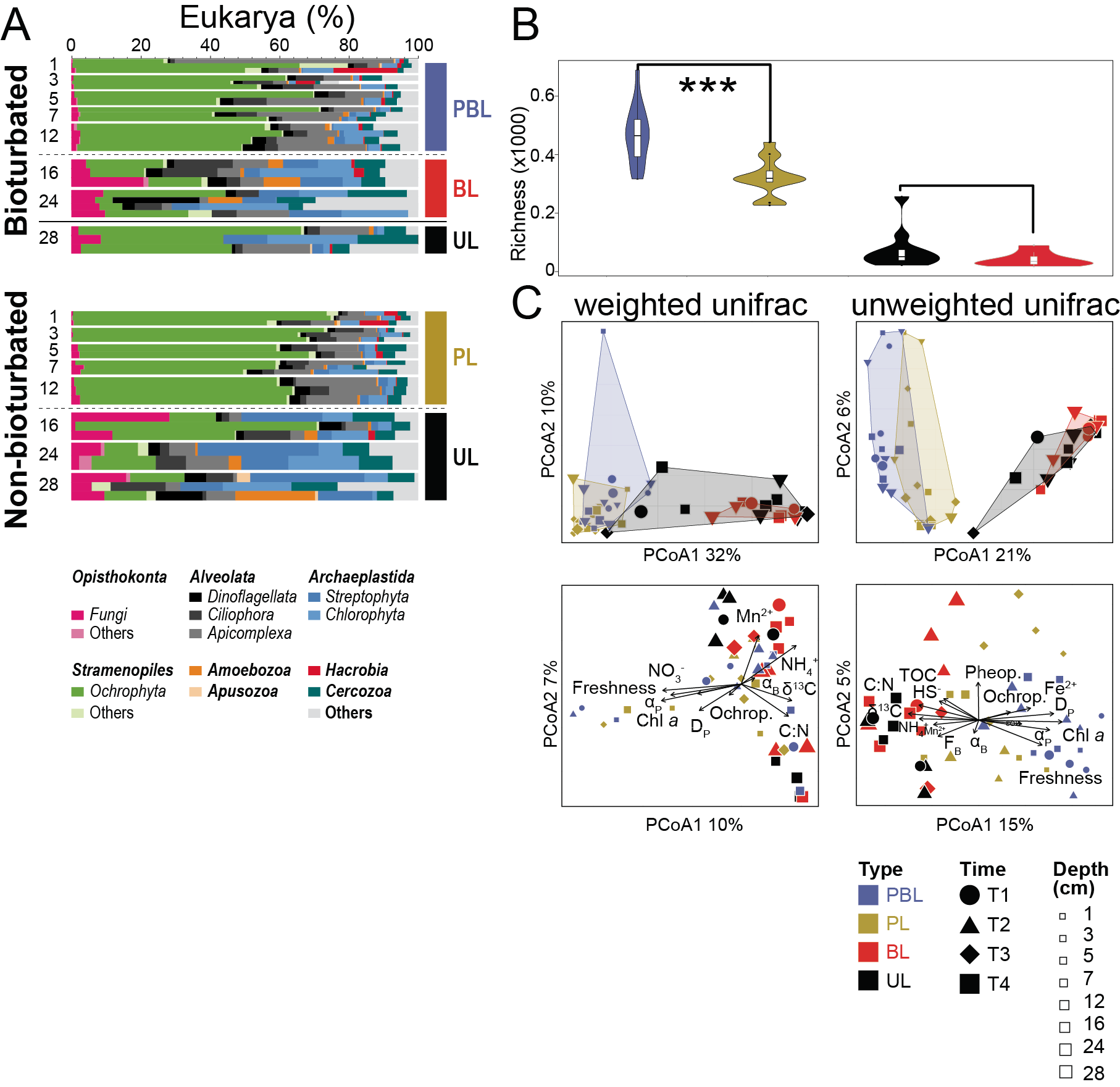


**Fig. S2. Community analyses on eukaryotic community with Metazoan sequences excluded.** (A) Relative abundances of dominant taxa at the phylum or class level across bioturbated vs. non-bioturbated sediments from all sampling dates (T1-T4) and sediment layers (1-28 cm, the numbers on y axis indicate sediment depth (cm)) (note: at each depth, there are three bars that are based on samplings on different dates). (B) Boxplots of richness. Asterisks denote significant differences (*p*<0.001) based on Welch’s t test. Community structuring based on (C) Weighted and unweighted Unifrac distance-based Principal Coordinates Analysis (PCoA) and Canonical Analysis of Principal Coordinates (CAP) in relation to environmental variables. All calculations in (B)-(C) were done based on zero-radius operational taxonomic units and involved all sampling dates. Only variables that were significantly correlated with community compositions (PERMANOVA *p*<0.05) are shown in CAP. [Abbreviations: PBL=physically and biologically impacted layer, PL=physically impacted layer, BL=biologically impacted layer, UL=undisturbed layer.]


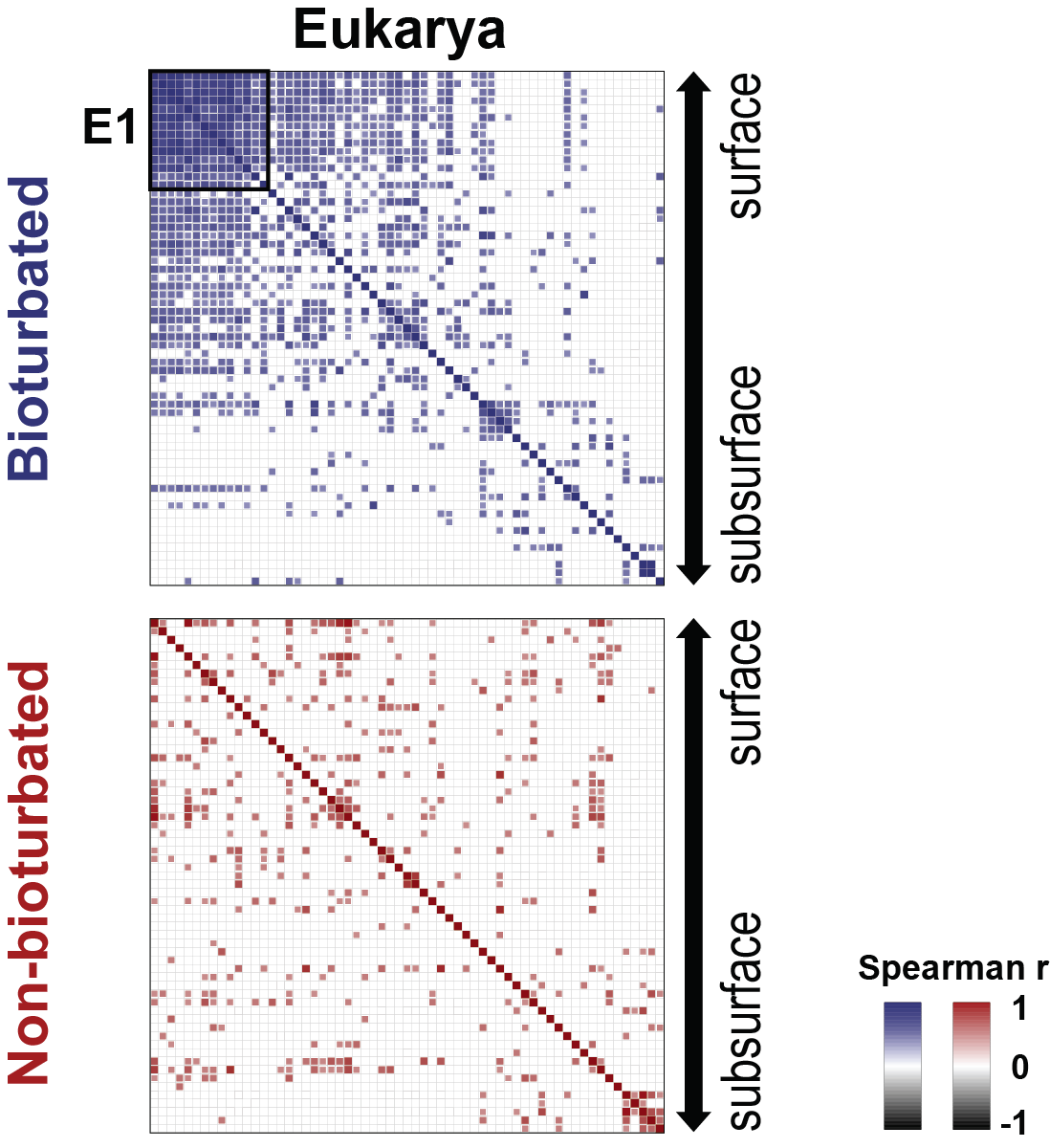


**Fig. S3. Correlation network analyses on eukaryotic communities with Metazoan sequences excluded.** Only pairwise Spearman correlations at the class-level with *p*<0.05 are shown. Classes are shown in the same vertical and horizontal order for bioturbation and non-bioturbated sediments. Cluster E1 was determined based on a hierarchical cluster analysis.

**
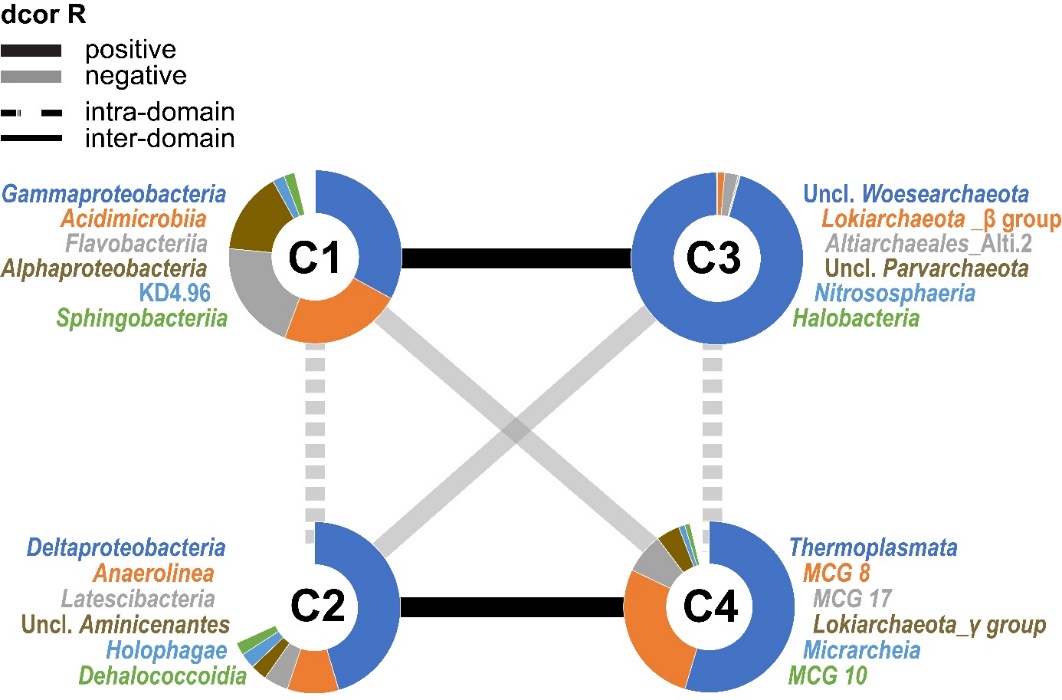
**

**Fig. S4.** Distance correlation test on clusters (C1-C4, see Fig. 5) in lugworm-free non-bioturbated sediments (999 bootstrap calculations). Only significant correlations (*p*<0.05) are reported.

**
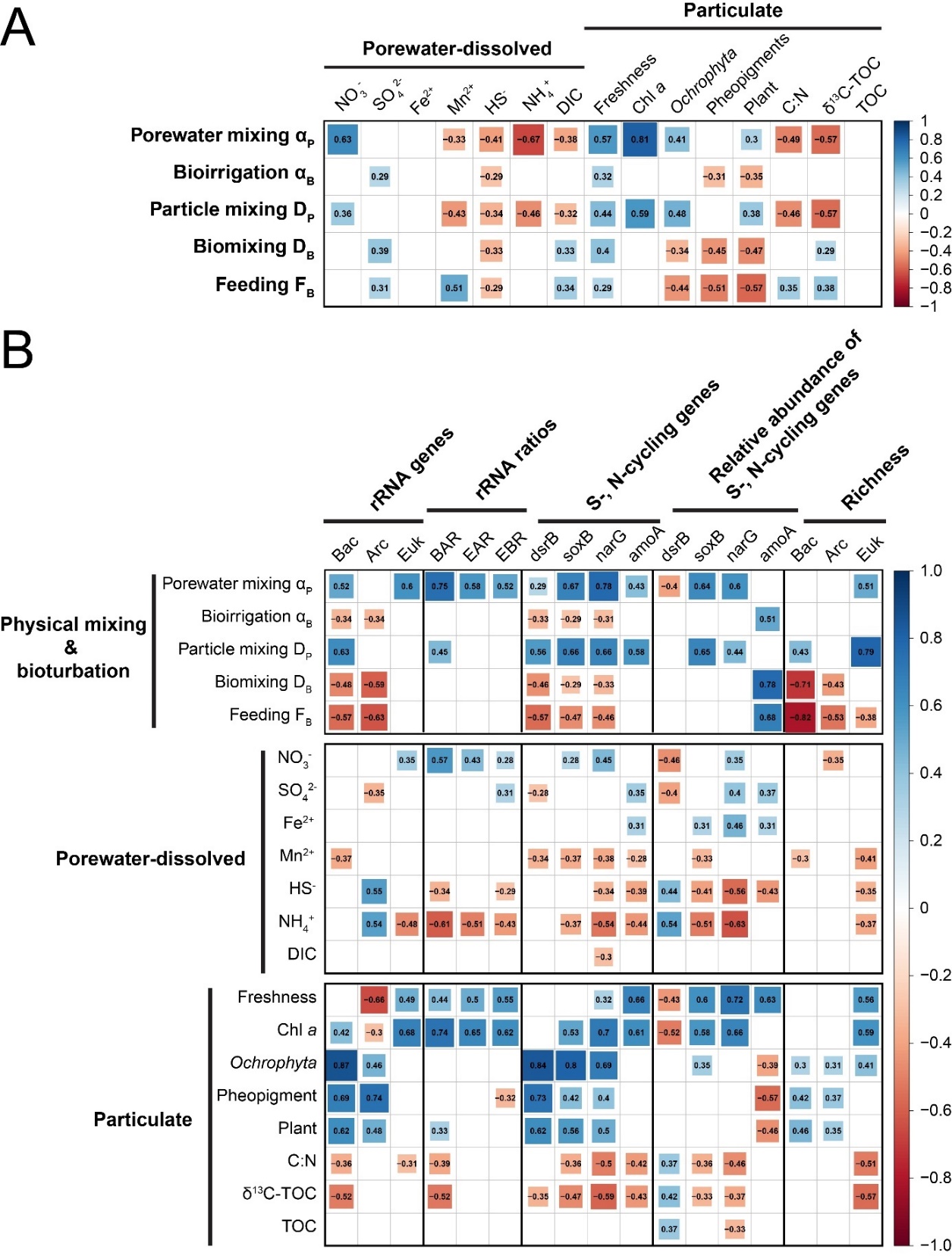
**

**Fig. S5.** Heatmap of relationships between (A) modeled mixing coefficients vs. geochemical data, and (B) modeled mixing coefficients and geochemical data vs. absolute and relative organismal gene abundances and ZOTU richness. All correlation values were calculated based on Pearson correlations, with r denoting the Pearson correlation coefficient, blue fields indicating significant positive correlations, and red fields indicating significant negative correlations. Blank fields indicate no significant correlation (p>0.05).


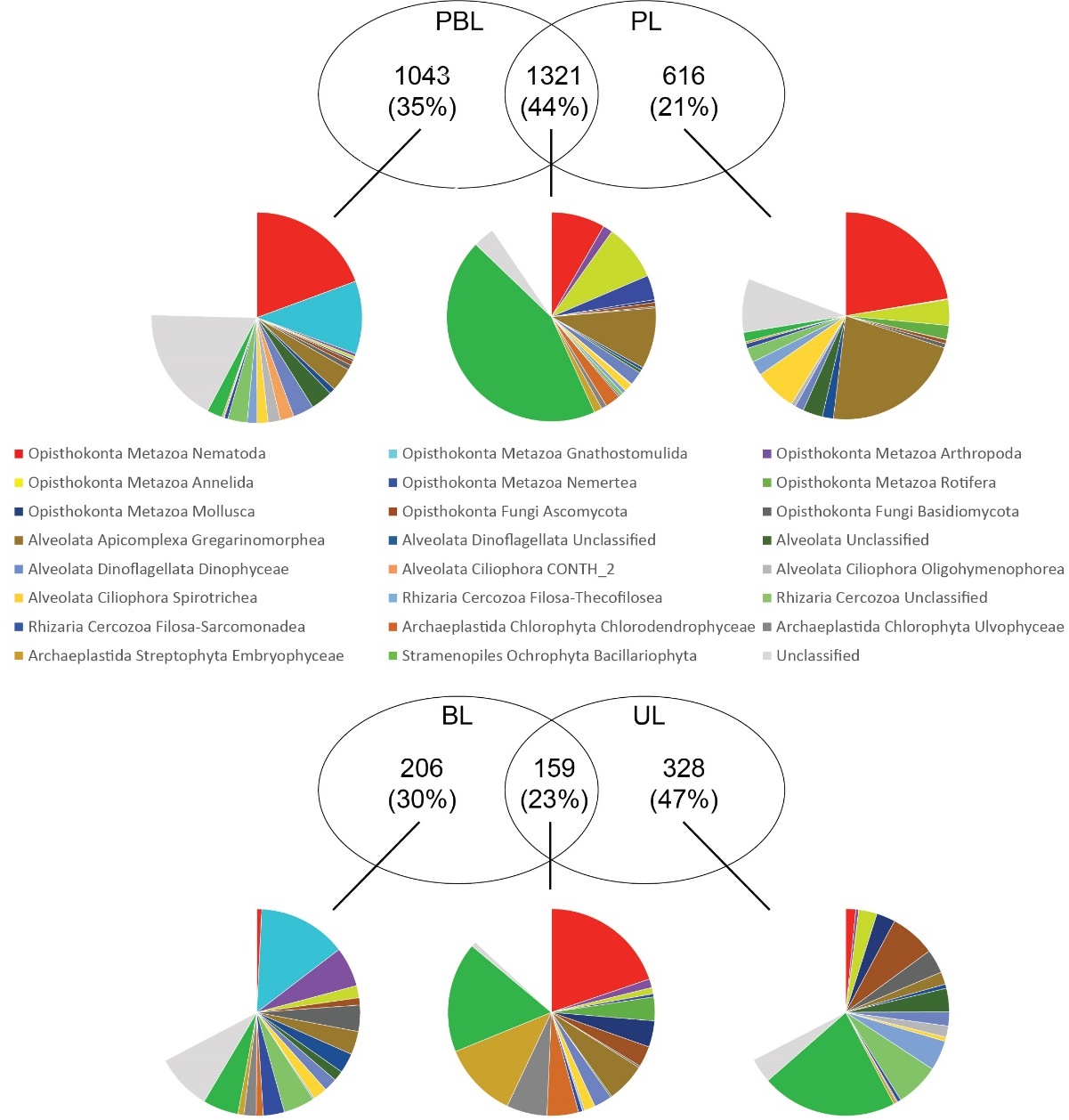


**Fig. S6.** Eukaryotic taxa that are unique to different sample types: physically and biologically impacted layers (PBL), physically impacted layers (PL), biologically impacted layers (BL), and undisturbed layers (UL).


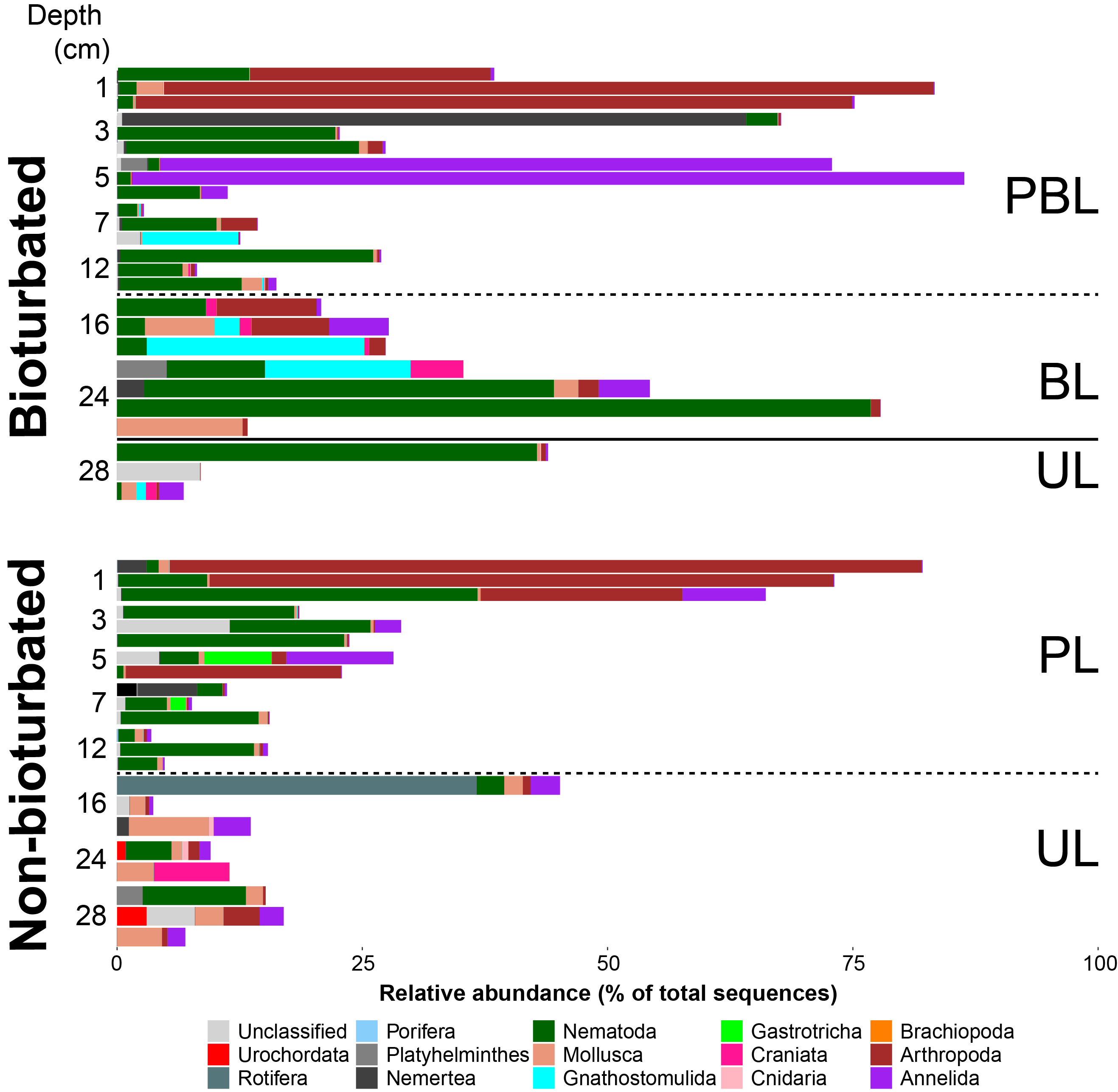


**Fig. S7.** Barcharts of relative abundances of Metazoan taxa versus sediment depth.


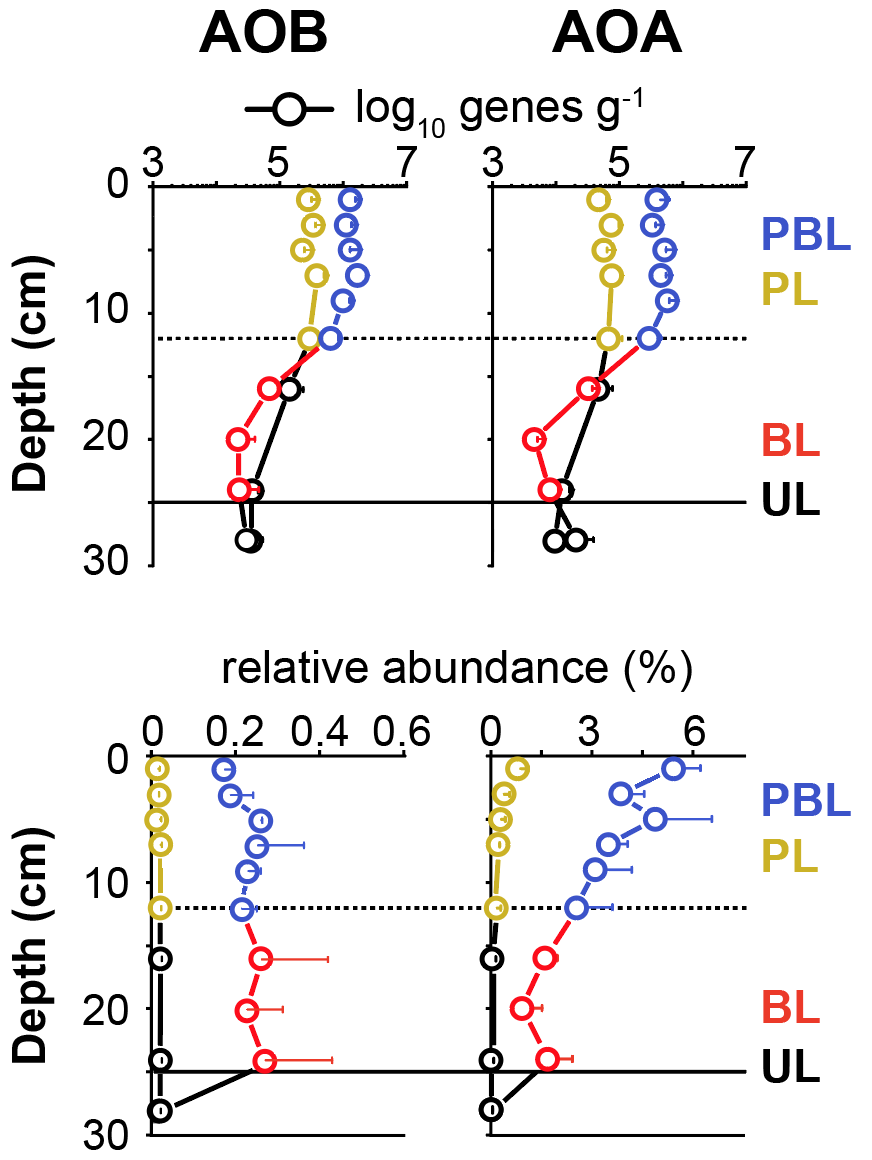


**Fig. S8.** Depth profiles of amoA gene copies that indicate the distributions of ammonium-oxidizing Bacteria (AOB) and ammonium-oxidizing Archaea (AOA) in sediment. Relative abundances of these genes in %, were calculated by dividing *amoA* gene copies by corresponding total 16S rRNA gene copy numbers. All values represent averages from three plots that were sampled at different time points (error bars denote standard deviations). [Abbreviations: PBL=physically and biologically impacted layer, PL=physically impacted layer, BL=biologically impacted layer, UL=undisturbed layer.]


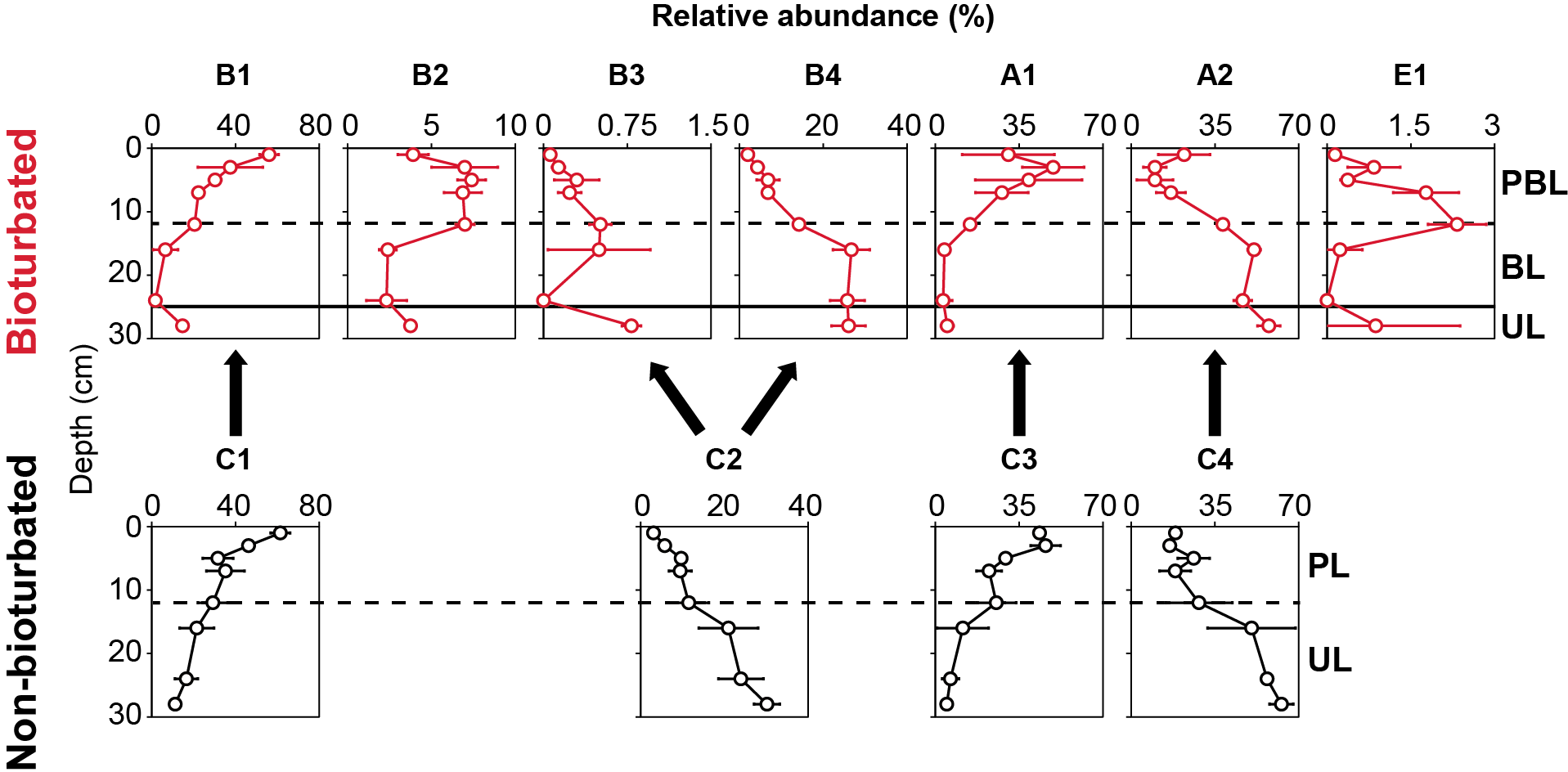


**Fig. S9.** Relative abundances of major network clusters plotted versus sediment depth.


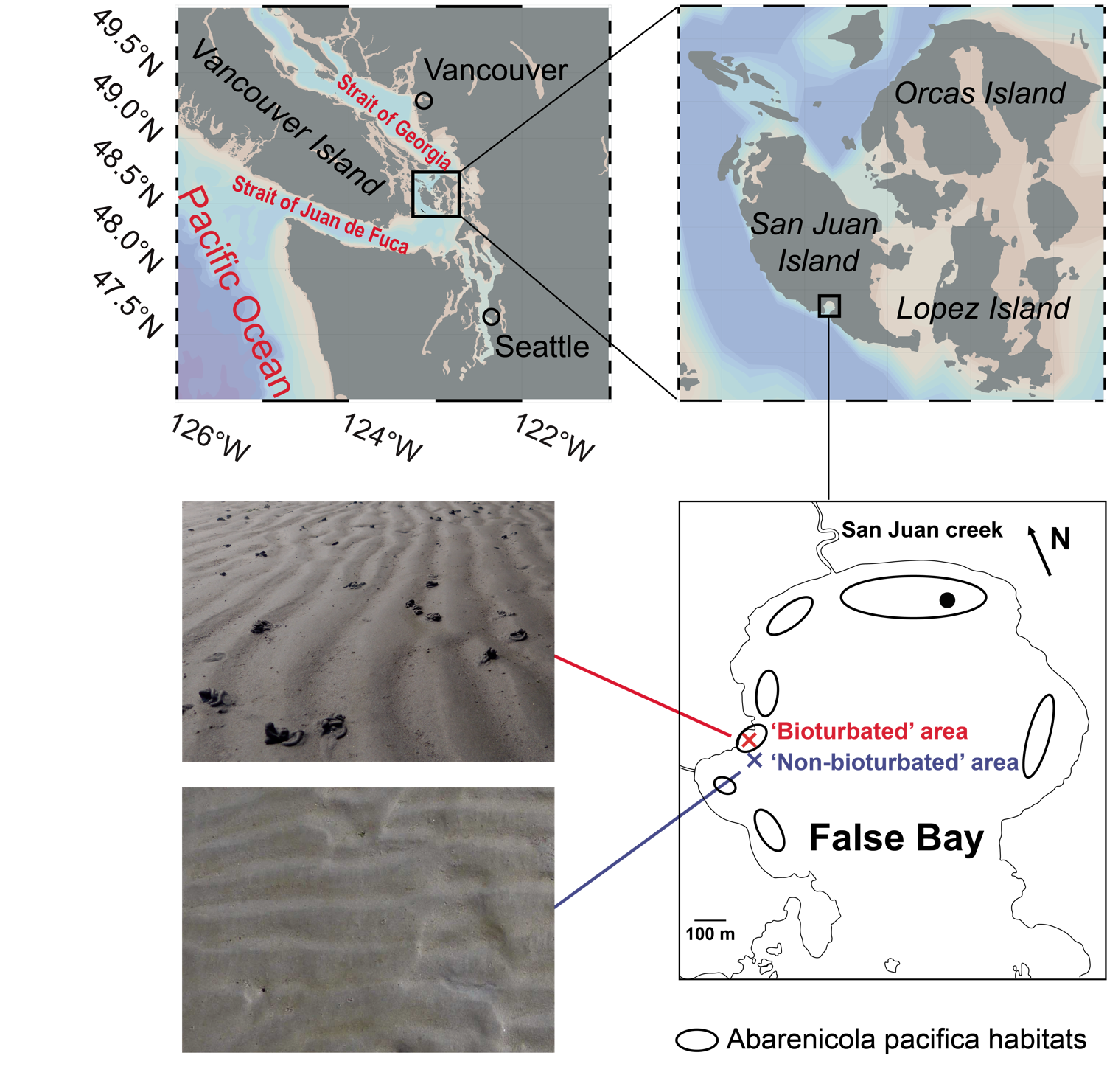


**Fig. S10.** Aerial maps and photos of sampling locations. The ‘Non-bioturbated’ area was *A. pacifica*-free, few small burrows (3-4 burrows m^-2^) were found in this area, but no fauna was captured when we dug up these burrows. The black dot indicates the site of a previous study on the influence of mixed communities of *A. pacifica* and thalassinid shrimps on porewater geochemical gradients (Waldbusser and Marinelli 2006).

**
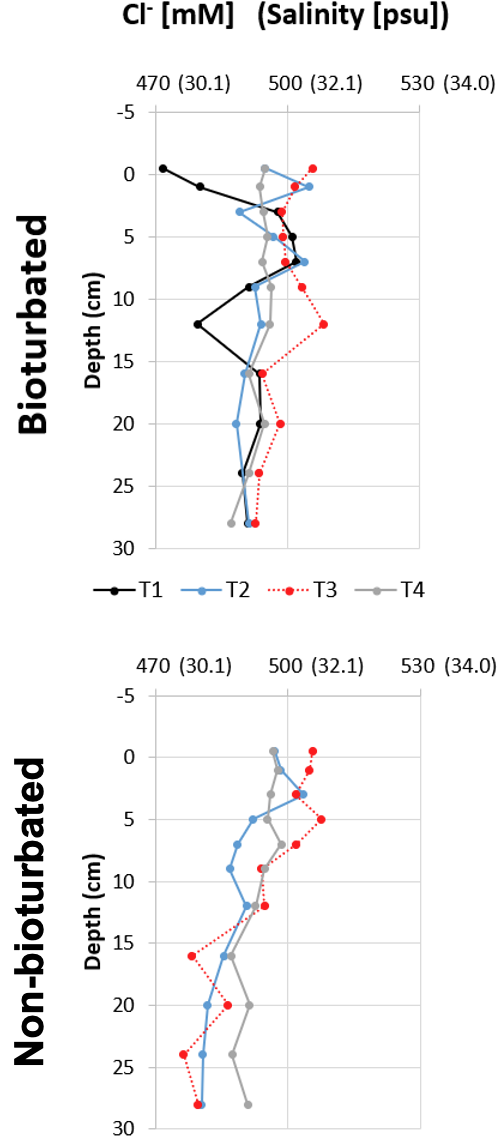
**

**Fig. S11.** Depth profiles of Cl^-^ concentration in bioturbated and non-bioturbated sediments. Values in parentheses are salinities calculated from Cl^-^ concentrations using the formula: Salinity (ppt) = Chlorinity (ppt) × 1.80655**.**


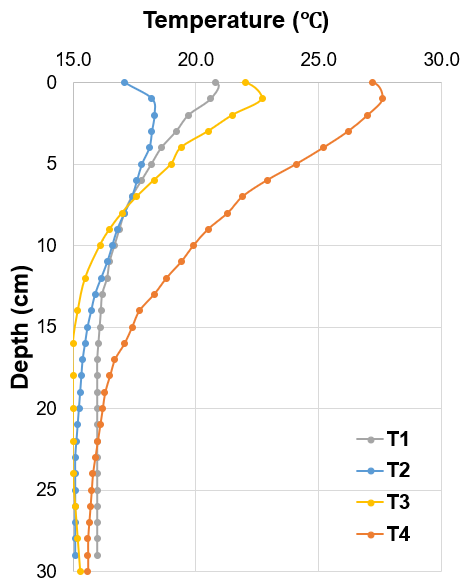


**Fig. S12.** Vertical temperature profiles in sediments before each sampling event (T1-T4).


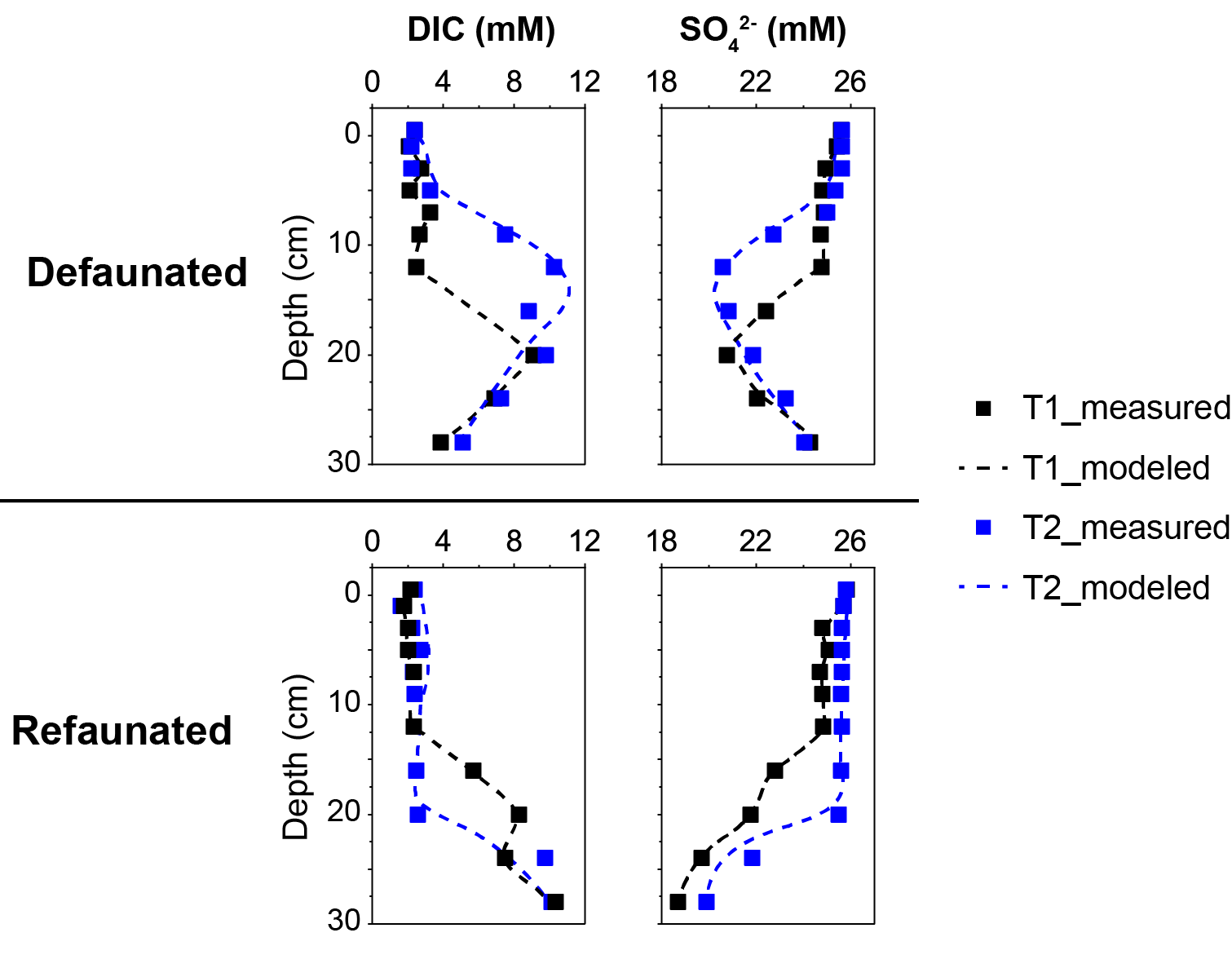


**Fig. S13.** Measured and modeled profiles of DIC and SO_4_^2-^ in the defaunated treatment (mimic ‘non-bioturbated’ sediments) and refaunated treatment (mimic ‘Bioturbated’ sediments). Modeled profiles were simulated using equation (S1) from “Modeled rates of porewater exchange by physical forcing and bioirrigation” in the Supplementary Text. Sulfate profiles were simulated by using a DIC production to sulfate consumption stoichiometry of 2:1. The latter assumes that dissimilatory sulfate reduction is the dominant mineralization process below the depth of O_2_ penetration.


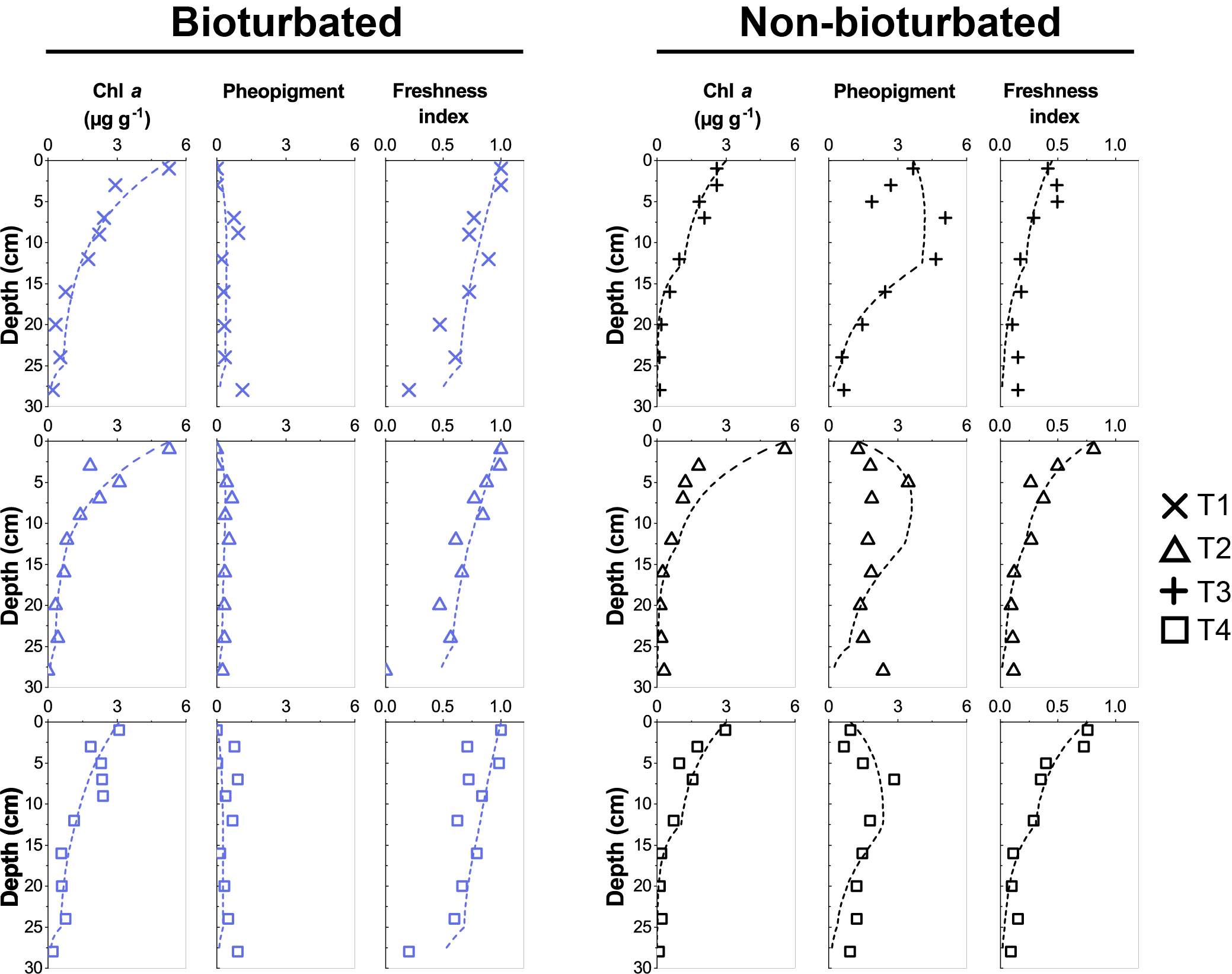


**Fig. S14.** Measured and modeled profiles of chl *a*, pheopigment, and freshness index in lugworm-inhabited (bioturbated) and lugworm-free (non-bioturbated) sediments. The modeled profiles were simulated using equations (2) and (3) from “Modeled rates of sediment mixing by physical and biological forcing” in the Supplementary Text.

Table S1 (*see next page*). Overview of the primers and standards used for the qPCR and sequencing assays. The *sox*B-1 primer combination was designed in this study after published primer combinations (*sox*B1164F/1446B and *sox*B693F/1164B (26); soxB1164F + *sox*B1446BK143 (27); phylotype-specific primer pairs (28)) did not produce clear or specific PCR amplicon bands. We designed low-degeneracy primer mixtures targeting nucleotide positions 521, 837, and 1170 within the *sox*B gene. Among these the primer mixture *sox*B-1, which targets the 837 and 1170 nucleotide positions, worked best. This was evidenced by the production of 1 clean, correctly-sized amplicon in DNA extracts from the samples studied, and the generation of a linear standard curve based on *sox*B qPCR standards of *Thiobacillus denitrificans*. These qPCR standards consisted of PCR products that had been produced using the *sox*B432F/1446R primer pair of ref. 26 and were confirmed by Sanger sequencing.

| **Target** | **Primer** | **Purpose** | **Sequence 5' - 3'** | **Approximate target length (bp)** | **Annealing Temp.**  **(℃)** | **Reference** | **Pure cultures for standards** |
| --- | --- | --- | --- | --- | --- | --- | --- |
| **Archaeal** | Arc915F_mod | qPCR | AAT TGG CGG GGG AGC AC | 144 | 55 | 13 | *Thermoplasma* |
| **16S rRNA** | Arc1059R |  | GCC ATG CAC CWC CTC T |  |  | 14 | *acidophilum* |
| **Bacterial** | Bac908F_mod | qPCR | AAC TCA AAK GAA TTG ACG GG | 167 | 60 | 15, 16 | *Desolfotignum* |
| **16S rRNA** | Bac1075R |  | CAC GAG CTG ACG ACA RCC |  |  | 16 | *phosphitoxidans* |
| **Eukarya** | All18SF_mod1 | qPCR | TGC ATG GCC GTT CTT AGT | 173 | 55 | 17 | *Tubifex tubifex* |
| **18S rRNA** | All18SR_mod1 |  | CTA AGG GCA TCA CAG ACC |  |  | 17 |  |
| **Ochrophyta**  **rbcL** | Ochro-rbcL_43F | qPCR | CGT TAC GAA TCT GGT GTA AT | 389 | 55 | 18 | *Stephanodiscus* sp. |
|  | Ochro-rbcL_432R |  | GGA ATA CGC ATA TCT TCT AAA CGT A |  |  | 18 |  |
| **Vascular** | rbcL h1aF | qPCR | GGC AGC ATT CCG AGT AAC TCC TC | 130 | 55 | 19 | *Salix* |
| **plant rbcL** | rbcL h2aR |  | CGT CCT TTG TAA CGA TCA AG |  |  | 19 | *chaenomeloides* |
| **narG** | NarG_1960F | qPCR | TAY GTS GGG CAG GAR AAA CTG | 90 | 60 | 20 | *Pseudomonas aeruginosa* |
|  | NarG_2050R |  | CGT AGA AGA AGC TGG TGC TGT T |  |  | 20 |  |
| **dsrB** | dsrB F1a-h | qPCR | CAC ACC CAG GGC TGG  CAT ACT CAG GGC TGG  CAT ACC CAG GGC TGG  CAC ACT CAA GGT TGG  CAC ACA CAG GGA TGG  CAC ACG CAG GGA TGG  CAC ACG CAG GGG TGG  CAT ACG CAA GGT TGG | 362 | 56 | 21 | *Desulfobulbus propionicus* |
|  | dsrB 4RSI1a-f |  | CAG TTA CCG CAG TAC AT  CAG TTA CCG CAG AAC AT  CAG TTG CCG CAG TAC AT  CAG TTT CCG CAG TAC AT  CAG TTG CCG CAG AAC AT  CAG TTT CCA CAG AAC AT |  |  | 21 |  |
| **soxB-1** | soxB-837Fa-i | qPCR | CAC AAC GGC ATG GAT GTN GA  CAC AAC GGC ATG GAC GTN GA  CAY AAC GGC TTC GAC GTS GA  CAY AAT GGC TTT GAC GTV GA  CAC AAC GGC TTT GAC GTV GA  CAT AAC GGC ATG GAT GTG GA  CAT GAT GGT TTT AGT GTT GA  CAT AAC GGC ATG CCG GTC GA  CAT GAT GGA TTC TCT GTG GA  CAC AAT GGT GCC GAT GTC GA  CAT AAC GGT ATG GAT GTT GA  CAT GAC GGG TTT GAC GTC GA | 333 | 60 | This study | *Thiobacillus denitrificans* |
|  | soxB-1170Ra-g |  | TT GAA RTT GCC SCG SCG RTA  TA GAA RGT ATC TCT TTT RTA  TA GAA ATT GTT GCG CCG RTA  TT AAA ATT ACC GCG TCG ATA  TC AAA ATT TCC CCG GCG ATA  TT AAA GTT GCC ACG ACG GTA  AA AAA TGT ATC ACG CTT ATA |  |  | This study |  |
| **amoA (AOA)** | Arch-amoAF | qPCR | STA ATG GTC TGG CTT AGA CG | 635 | 53 | 22 | *Nitrososphaera viennensis* |
|  | Arch-amoAR |  | GCG GCC ATC CAT CTG TAT GT |  |  | 22 |  |
| **amoA (AOB)** | amoA-1F | qPCR | GGG GTT TCT ACT GGT GGT | 491 | 55.4 | 23 | *Nitrosomonas europaea* |
|  | amoA-2R KS |  | CCC CTC KGS AAA GCC TTC TTC |  |  | 23 |  |
| **Archaeal** | ARC519F | Sequencing | CAG CMG CCG CGG TAA HAC C | 396 | 63 | 24 |  |
| **16S rRNA** | ARC 915Rmod |  | GTG CTC CCC CGC CAA TT |  |  | 13 |  |
| **Bacterial** | S-D-Bact-0341-b-S-17 | Sequencing | CCT ACG GGN GGC WGC AG | 444 | 50-55 | 25 |  |
| **16S rRNA** | S-D-Bact-0785-a-A-21 |  | GAC TAC HVG GGT ATC TAA TCC |  |  | 25 |  |
| **Eukarya** | All18SF_mod1 | Sequencing | TGC ATG GCC GTT CTT AGT | 173 | 55 | 17 |  |
| **18S rRNA** | All18SR_mod1 |  | CTA AGG GCA TCA CAG ACC |  |  | 17 |  |

Table S2. Temperature protocols and corresponding time intervals for the qPCR assays.

| **qPCR step** | **Archaea 16S** | **Bacteria 16S** | **Eukarya 18S** | ***Ochrophyta rbc*L** | **Vascular plant rbcL** | ***nar*G** | ***dsr*B** | ***sox*B** | ***amo*A (AOA)** | ***amo*A**  **(AOB)** |
| --- | --- | --- | --- | --- | --- | --- | --- | --- | --- | --- |
|  | Time: min:ss  (Temperature: ℃) | | | | | | | | | |
| **1. Activation** | 05:00  (95) | 05:00  (95) | 05:00  (95) | 05:00  (95) | 05:00  (95) | 05:00  (95) | 05:00  (95) | 05:00  (95) | 10:00  (95) | 10:00  (95) |
| **2. Denaturation** | 00:10  (95) | 00:10  (95) | 00:10  (95) | 00:30  (95) | 00:30  (95) | 00:30  (95) | 00:30  (95) | 00:10  (95) | 00:30  (95) | 00:30  (95) |
| **3. Annealing** | 00:30  (55) | 00:30  (60) | 00:30  (55) | 00:40  (55) | 00:40  (55) | 00:30  (60) | 00:30  (56) | 00:30  (60) | 00:45  (53) | 01:00  (55.4) |
| **4. Polymerization** | 00:15  (72) | 00:15  (72) | 00:20  (72) | 00:30  (72) | 00:30  (72) | 00:15  (72) | 00:20  (72) | 00:15  (72) | 00:55  (72) | 00:55  (72) |
| **5. Acquisition** | 00:05  (80) | 00:05  (80) | 00:05  (80) | 00:05  (78) | 00:05  (80) | 00:05  (72) | 00:05  (82) | 00:05  (82) | 00:05  (72) | 00:05  (72) |
| **Repeat step 2-5: 50 cycles** | | | | | | | | | | |
| **6. Melting curve** | 01:00  (95) | | | | | | | | | |
|  | 1°C min^-1^  (60-95) | 1°C min^-1^  (60-95) | 1°C min^-1^  (55-95) | 1°C min^-1^  (55-95) | 1°C min^-1^  (55-95) | 1°C min^-1^  (65-95) | 1°C min^-1^  (55-95) | 1°C min^-1^  (55-95) | 1°C min^-1^  (55-95) | 1°C min^-1^  (55-95) |

**References**

1. E. Kristensen, Impact of polychaetes (Nereis spp. and Arenicola marina) on carbon biogeochemistry in coastal marine sediments, *Geochemical Transactions* **2**(12), 92-103 (2001).
2. B. P. Boudreau, Diagenetic models and their implementation (Springer, Berlin, 1997).
3. M. Huettel, P. Berg, J. E. Kostka, Benthic exchange and biogeochemical cycling in permeable sediments. *Ann. Rev. Mar. Sci.* 6:23-51 (2014).
4. V. C. Philippe, Y. Wang, Cycling of iron and manganese in surface sediments; a general theory for the coupled transport and reaction of carbon, oxygen, nitrogen, sulfur, iron, and manganese. *American Journal of Science* **296**(3), 197-243 (1996).
5. F. J. R. Meysman, O. S. Galaktionov, B. Gribsholt, J. J. Middelburg, Bioirrigation in permeable sediments: Advective pore‐water transport induced by burrow ventilation. *Limnol. Oceanogr.* **51**(1), 142-156 (2006).
6. T. M. Dornhoffer, G. G. Waldbusser, C. Meile, Modeling lugworm irrigation behavior effects on sediment nitrogen cycling. Mar. Ecol. Prog. Ser. 534: 121-134 (2015).
7. E. Kristensen, H. Røy, K. Debrabant, T. Valdemarsen, Carbon oxidation and bioirrigation in sediments along a Skagerrak-Kattegat-Belt Sea depth transect. *Mar. Ecol. Prog. Ser.* *604*, 33-50 (2018).
8. M. A. Green, J. D. Gulnick, N. Dowse, P. Chapman, Spatiotemporal patterns of carbon remineralization and bio-irrigation in sediments of Casco Bay Estuary, Gulf of Maine. *Limnol. Oceanogr.* ***49***(2), 396-407 (2004).
9. M. Y. Sun, R. C. Aller, C. Lee, Early diagenesis of chlorophyll-*a* in Long Island Sound sediments: A measure of carbon flux and particle reworking. *J. Mar. Res*. **49**(2), 379-401 (1991).
10. M. Y. Sun, R. C. Aller, C. Lee, Spatial and temporal distributions of sedimentary chloropigments as indicators of benthic processes in Long Island Sound. *J. Mar. Res.* **52**(1), 149-176 (1994).
11. A. E. Ingalls, R. C. Aller, C. Lee, M. Y. Sun, The influence of deposit-feeding on chlorophyll-a degradation in coastal marine sediments. *J. Mar. Res.* **58**(4), 631-651 (2000).
12. E. T. Furlong, C. Roy, Pigment preservation and remineralization in oxic coastal marine sediments. *Geochim. Cosmochim. Acta.* **52**(1), 87-99 (1988).
13. H. Cadillo-Quiroz, S. Bräuer, E. Yashiro, C. Sun, J. Yavitt, S. Zinder, Vertical profiles of methanogenesis and methanogens in two contrasting acidic peatlands in central New York State, USA. *Environ. Microbiol.* **8**(8), 1428-1440 (2006).
14. Y. Yu, C. Lee, J. Kim, S. Hwang, Group-specific primer and probe sets to detect methanogenic communities using quantitative real-time polymerase chain reaction. *Biotechnol. Bioeng.* **89**(6), 670-679 (2005).
15. M. A. Lever, A. Torti, P. Eickenbusch, A. B. Michaud, T. Šantl-Temkiv, B. B. Jørgensen, A modular method for the extraction of DNA and RNA, and the separation of DNA pools from diverse environmental sample types. *Front. Microbiol.* **6**, 1-25 (2015).
16. M. Ohkuma, T. Kudo, Phylogenetic analysis of the symbiotic intestinal microflora of the termite Cryptotermes domesticus. *FEMS Microbiol. Lett.* **164**(2), 389–395 (1998).
17. A. Torti, “Extraction and phylogenetic survey of extracellular and intracellular DNA in marine sediments”, thesis, Aarhus University, Denmark (2015).
18. X. Han J. Tolu, L. Deng, A. Fiskal, C. Schubert, L. Winkel, M. Lever, Long-term preservation of biomolecules in lake sediments: potential importance of physical shielding. *In press.*
19. E. Willerslev, A. J. Hansen, J. Binladen, T. B. Brand, M. T. P. Gilbert, B. Shapiro, M. Bunce, C. Wiuf, D. A. Gilichinsky, A. Cooper, Diverse plant and animal genetic records from Holocene and Pleistocene sediments. *Science* **300**(5620), 791-795 (2003).
20. J. C. López-Gutiérrez, S. Henry, S. Hallet, F. Martin-Laurent, G. Catroux, L. Philippot, Quantification of a novel group of nitrate-reducing bacteria in the environment by real-time PCR. *J. Microbiol. Methods* 57(3) (2004): 399-407.
21. M. A. Lever, O. Rouxel, J. C. Alt, N. Shimizu, S. Ono, R. M. Coggon, W. C. Shanks III, L. Lapham, M. Elvert, A. Teske, Evidence for microbial carbon and sulfur cycling in deeply buried ridge flank basalt. *Science* **339**(6125), 1305-1308 (2013).
22. C. A. Francis, K. J. Roberts, J. M. Beman, A. E. Santoro, B. B. Oakley, Ubiquity and diversity of ammonia-oxidizing archaea in water columns and sediments of the ocean. *Proc. Natl. Acad. Sci. U.S.A.*, **102**(41), 14683-14688 (2005).
23. J. H. Rotthauwe, K. P. Witzel, W. Liesack, The ammonia monooxygenase structural gene *amo*A as a functional marker: molecular fine-scale analysis of natural ammonia-oxidizing populations. *Appl. Environ. Microbiol.* **63**(12), 4704-4712 (1997).
24. K. B. Sørensen, A. Teske, Stratified communities of active Archaea in deep marine subsurface sediments. *Appl. Environ. Microbiol.* **72**(7), 4596-4603 (2006).
25. D. P. Herlemann, M. Labrenz, K. Jürgens, S. Bertilsson, J. J. Waniek, A. F. Andersson, Transitions in bacterial communities along the 2000 km salinity gradient of the Baltic Sea. *ISME J.*, **5**(10), 1571-1579 (2011).
26. R. Petri, L. Podgorsek, J.F. Imhoff, Phylogeny and distribution of the *sox*B gene among thiosulfate-oxidizing bacteria. *FEMS Microbiol. Lett.*, **197**(2), 171-178 (2001).
27. K. K. Krishnani, V. Kathiravan, M. Natarajan, M. Kailasam, S. M. Pillai, Diversity of sulfur-oxidizing bacteria in greenwater system of coastal aquaculture. *Appl. Biochem. Biotechnol.* **162**, 1225-1237 (2010).
28. F. Thomas, A. E. Giblin, Z. G. Cardon, S. M. Sievert, Rhizosphere heterogeneity shapes abundance and activity of sulfur-oxidizing bacteria in vegetated salt marsh sediments. *Front. Microbiol.*, **5**, 309 (2014).
